# Age-related changes in oral sensitivity, taste and smell

**DOI:** 10.1038/s41598-022-05201-2

**Published:** 2022-01-27

**Authors:** Tobias Braun, Johanna M. Doerr, Laura Peters, Maxime Viard, Iris Reuter, Mario Prosiegel, Susanne Weber, Mesut Yeniguen, Marlene Tschernatsch, Tibo Gerriets, Martin Juenemann, Hagen B. Huttner, Samra Hamzic

**Affiliations:** 1 Department of Neurology, University Hospital Giessen and Marburg, Justus Liebig University, Klinikstrasse 33, 35392 Giessen, Germany; 2 Justus-Liebig-University Giessen, Faculty of Medicine, Klinikstrasse 29, 35392 Giessen, Germany; 3 Faculty of Languages and Literatures, Department I, Ludwig-Maximilians-University (LMU), Munich, Germany; 4 Stroke Unit, Buergerhospital Friedberg, Ockstaedter Str. 3-5, 61169 Friedberg, Germany; 5 Department of Neurology, Gesundheitszentrum Wetterau, Chaumontplatz 1, 61231 Bad Nauheim, Germany

**Keywords:** Gustatory system, Olfactory system, Ageing

## Abstract

Oropharyngeal sensitivity plays a vital role in the initiation of the swallowing reflex and is thought to decline as part of the aging-process. Taste and smell functions appear to decline with age as well. The aim of our study was to generate data of oral sensitivity in healthy participants for future studies and to analyse age-related changes and their interdependence by measuring oral sensitivity, taste, and smell function. The experiment involved 30 participants younger than and 30 participants older than 60. Sensitivity threshold as a surrogate of oral sensitivity was measured at the anterior faucial pillar by electrical stimulation using commercially available pudendal electrode mounted on a gloved finger. Smell and taste were evaluated using commercially available test kits. Mean sensitivity was lower in young participants compared to older participants (1.9 ± 0.59 mA vs. 2.42 ± 1.03 mA; *p* = 0.021). Young participants also performed better in smell (Score 11.13 ± 0.86 vs 9.3 ± 1.93; *p* < 0.001) and taste examinations (Score 11.83 ± 1.86 vs 8.53 ± 3.18; *p* < 0.001). ANCOVA revealed a statistical association between sensitivity and smell (*p* = 0.08) that was moderated by age (*p* = 0.044). Electrical threshold testing at the anterior faucial pillar is a simple, safe, and accurate diagnostic measure of oral sensitivity. We detected a decline of oral sensitivity, taste, and smell in older adults.

**Trial registration:** Clinicaltrials.gov, NCT03240965. Registered 7th August 2017—https://clinicaltrials.gov/ct2/show/NCT03240965.

## Introduction

Dysphagia occurs in various diseases and clinical specialties, especially in neurological patients. It is associated with increased mortality and morbidity, as well as longer hospital stay and high treatment costs^1,2^. In the acute phase of stroke up to 80% of patients are diagnosed with swallowing disorders^3^. The most serious consequence of dysphagia after stroke is aspiration pneumonia. Pneumonia causes the highest attributable mortality of all medical complications following stroke^4^. Therefore, dysphagia determines the immediate prognosis and is of relevance to neurological patients due to the functional link to the central and peripheral nervous system.

Although research in the field of dysphagia is rising, there are still large gaps in our understanding of swallowing and its central nervous control.

Scientific literature on sensory functions in normal and disordered swallowing is heterogeneous. Research varies in the degree of importance given to various oropharyngeal regions such as mucosa of the tongue base, faucial pillars, valleculae, epiglottis, or laryngeal folds in the swallowing process. The heterogeneity stems from several experiments in human subjects (healthy controls or patients) or different animals, methods for measurement of sensory function and mode of swallowing (voluntary vs. involuntary)^5^. The mode of swallowing in particular is believed to have a large effect on swallowing function because voluntary swallowing appears to be entirely different from involuntary swallowing^6–9^. Furthermore, taste and smell appear to play an important role in swallowing as well, because latency of swallowing reflex is reduced when olfactory and gustatory information are presented^5,10–12^.

Oropharyngeal sensitivity, taste and smell deteriorate during life as part of the normal ageing process intensely^13–16^. Mortelliti et al. found a lower density of myelinated fibres in the superior laryngeal nerve in old adults compared to young people in an autopsy study, which might explain the loss of sensitivity^17^. A delayed swallowing reflex is attributed to reduced oropharyngeal sensitivity^18^ and is often found in older patients. These age-related changes can impair the swallowing function, which was referred to “presbyphagia”^16^. Presbyphagia does not necessarily lead to penetration and aspiration of food and fluids, but it might limit patients’ compensatory abilities in case of an acute impairment of swallowing function (ie., due to acute stroke)^16^.

Various approaches to assessing oral sensory function already exist. Stimulation of oropharyngeal regions using air puffs, testing with Frey filaments, or simple bilateral testing using a cotton swab have been described earlier^19,20^. Power et al. described an objective and quantifiable approach using electrical stimulation of oral structures with an electrode mounted on a gloved finger^21^.

When designing a trial to assess sensory function in a large cohort of stroke patients using the method described by Power et al., the first step was generating data from healthy controls with a similar range of age as stroke patients (older than 59 years). Younger participants underwent the procedure first for verification of the model used. The results from the experiment in younger adults were recently published and were used in this experiment as control^22^. The data from older adults gave us the opportunity to evaluate age-related effects on oral sensitivity, taste, and smell function and their interdependence. We hypothesized that deterioration of oral sensitivity in older participants could be demonstrated by this approach. By measuring the smell and taste function, we analysed the interdependence and the effect of age on all 3 sensory modalities (oral sensitivity, taste and smell) by using analysis of covariance (ANCOVA) and a moderation analysis.

The sensory threshold was examined at the anterior faucial pillar. This region is of particular interest because it is the most distal area of the oropharyngeal tract that can be reached easily with the examiner’s finger and without side effects such as coughing or gagging. The faucial area is assumed to play a significant role in swallowing. Projections via the glossopharyngeal nerve from each faucial pillar to the sensory cortex of both hemispheres with ipsilateral predominance were identified^23^. Data suggest that the contact of the bolus with the faucial pillar triggers the swallowing reflex^24–26^, although some authors did not find evidence for this theory^27,28^. The theory stems from the observation that the elevation of the hyoid, which marks the beginning of pharyngeal swallowing, is initiated when the bolus contacts the faucial pillar region^29,30^.

## Results

### Participants’ characteristics

In the group of older adults, 5 participants withdrew their consent after initial consent and no data were acquired. No difference in sex or smoking status was observed between groups (see Table 1).

**Table 1:** Descriptive values of age, sex, and smoking status.

	Younger participants (age < 60) (*n* = 30)	Older participants (*n* = 30)	*p*-value
Age (mean [SD])	27 (5.1)	71.2 (8.4)	< 0.001
Male (*n* [%])	16 (53.3%)	13 (43.3%)	0.438
Cigarette smoking (*n* [%])	5 (16.7)	4 (13.3%)	0.718

The difference in age was assessed with independent *t* tests, the difference in the other (categorical) variables with χ^2^ tests.

### Between-group differences in sensitivity, taste, and smell

Taste and smell tests revealed that in the group of younger adults 2 participants (6.7%) were hypogeusic and 5 (16.7%) were hyposmic, whereas in the group of older adults 16 participants (53.3%) were hypogeusic and 21 (70%) were hyposmic. When controlled for sex and smoking status, the mean sensitivity threshold, *F*_(1, 58)_ = 6.02, *P* = 0.017, η^2^ = 0.10 (medium to large effect); taste score, *F*_(1, 58)_ = 27.63, *P* < 0.001, η^2^ = 0.33 (large effect); and smell score, *F*_(1, 58)_ = 24.04, *P* < 0.001, η^2^ = 0.30 (large effect) differed significantly between groups. This was also the case for every taste sub-score (sweet: *F*_(1, 58)_ = 17.21, *P* < 0.001, η^2^ = 0.24 (large effect); salty: *F*_(1, 58)_ = 5.30, *P* = 0.025, η^2^ = 0.09 (medium to large effect); sour: *F*_(1, 58)_ = 22.72, *P* < 0.001, η^2^ = 0.29 (large effect); bitter: *F*_(1, 58)_ = 10.90, *P* = 0.002, η^2^ = 0.16 (large effect)). Post hoc *t* tests for independent samples confirm these effects, showing a higher sensitivity threshold and lower taste and smell scores in the older adults group (see Table 2). A significant difference was also observed between sexes in taste score, *F*_(1, 58)_ = 5.01, *P* = 0.029, η^2^ = 0.08 (medium effect), showing a slightly higher taste score in women as compared to men when controlled for age group and smoking status. However, this effect was not statistically significant in a post hoc independent sample *t* test, *t*_(58)_ = 1.39, *P* = 0.171, suggesting it is dependent on controlling for age. Sensitivity threshold and smell score were both independent of sex and cigarette smoking status (data not shown).

**Table 2:** Differences in sensitivity thresholds and taste as well as smell scores between groups (*n* = 30 in each group).

	Young participants M (SD)	Older participants M (SD)	*p*-value
**Sensitivity threshold**			
Left side	1.88 mA (0.66)	2.42 mA (1.09)	0.025
Right side	1.88 mA (0.60)	2.33 mA (1.04)	0.048
Both sides (mean)	1.90 mA (0.59)	2.42 mA (1.03)	0.021
**Taste score**	11.83 (1.86)	8.53 (3.18)	< 0.001
Sweet	3.53 (0.73)	2.63 (1.10)	< 0.001
Salty	2.97 (0.89)	2.30 (1.26)	0.020
Sour	2.53 (0.57)	1.37 (1.16)	< 0.001
Bitter	2.80 (1.10)	1.90 (1.47)	0.010
Smell score	11.13 (0.86)	9.3 (1.93)	< 0.001

Differences between groups were assessed using independent *t* tests. No differences were observed between the sensitivity threshold of the left and right side in the young, *t*_(28)_ = 0.12, *P* = 0.904, or the older group, *t*_(28)_ = 0.21, *P* = 0.833.

### Associations between sensitivity, taste, and smell

When controlled for age group, sex, and smoking status, no significant association was found between sensitivity threshold and taste (sum score: *b* = 0.10, *t*_(4,55)_ = 0.65, *P* = 0.520; sweet:* b* = 0.16, *t*_(4,55)_ = 1.08, *P* = 0.284; salty: *b* = − 0.09, *t*_(4,55)_ = − 0.67, *P* = 0.504; sour:* b* = − 1.11, *t*_(4,55)_ =  − 0.75, *P* = 0.459; bitter:* b* = 0.12, *t*_(4,55)_ = 0.81, *P* = 0.423) or between taste and smell score (*b* = 0.09, *t*_(4,55)_ = 0.69, *P* = 0.495). There was, However, a statistically significant association between sensitivity threshold and smell score (*b* =  − 0.62, *t*_(4,55)_ =  − 2.77, *P* = 0.008), suggesting a 0.62 point decrease in smell score with every 1 unit increase in sensitivity threshold. The adjusted *R*^2^ for the overall model was 0.35, indicative of a high goodness-of-fit according to Cohen^31^.

### Moderating effect of age

**Figure 1:**
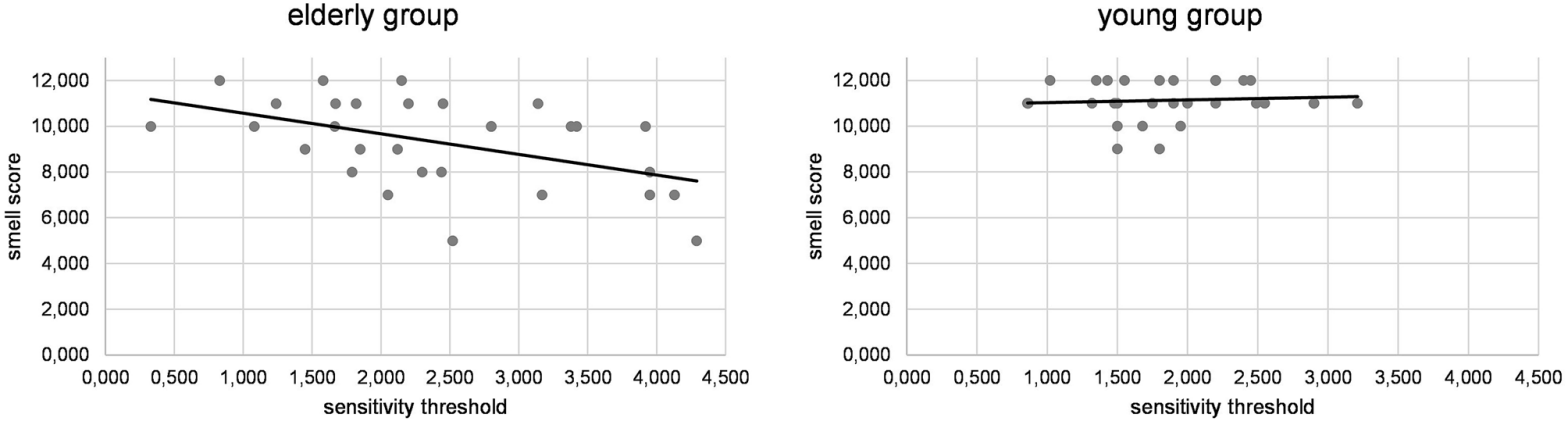
Association of smell score and sensitivity threshold (milliamps) in the group of young and older participants. The association was significant only in the group of older participants.

A moderation analysis was run to determine whether age had a significant effect on the association between sensitivity threshold and smell score. The overall model was significant, *F*(3, 56) = 13.55, *P* < 0.001, predicting 42.06% of the variance. Results showed that age group significantly moderated the effect between sensitivity threshold and smell score, Δ*R*^2^ = 4.39%, *F*(1, 56) = 4.25, *P* = 0.044 (95% CI  − 2.01 to − 0.03). Further analyses separated by group showed that the association between sensitivity threshold and smell was significant in the older group (*b* = − 0.90, *t*_(1,28)_ =  − 2.92, *P* = 0.007), but not in the young group (*b* = 0.12, *t*_(1,28)_ = 0.43, *P* = 0.671; see Fig. 1).

## Discussion

In the present study, we measured the oral sensitivity threshold in young and old healthy participants using electrical stimulation and assessed taste and smell function. Our results were analysed for age-related changes of the 3 items and their interdependence. By electrically stimulating of the anterior faucial pillars, we were able to quantify oral sensitivity in a safe and time-efficient manner. The test was well tolerated by all participants. Our data show a significant deterioration of oral sensitivity, taste (which did not differ between different taste qualities), and smell in older adults. A significant association of the smell function and the sensory threshold was moderated by age in the older group but not in the younger group.

A reduction of oropharyngeal sensitivity is often viewed as part of the normal aging process^32,33^. A reduction of myelinated nerve fibres of the oropharyngeal nerves is thought to be the underlying pathology^17^. Only a sparse number of studies have investigated this phenomenon in an objectifiable way. Touching oropharyngeal structures with a cotton swab or with a needle is a widely used but not quantifiable clinical routine. Nevertheless, these methods were solely used to describe age-related changes in oropharyngeal sensitivity in the past^34,35^. One semiquantifiable method is the use of monofilaments^36^. Our search of the literature did not identify studies that have used this method in healthy older adults. Shupe et al. proposed an approach in which participants had to identify letters or different shapes with their tongue^37^. Older adults performed worse than younger participants did in these tasks. However, this test is not limited solely to oral sensitivity; it also examines oral stereognosis, which represents a higher neuropsychological function of the brain. Labeit et al. proposed an oral-sensitivity test that involved delivering a small fluid bolus into the pharynx and measuring the latency of swallowing. The authors stated the test has limitations because the latency comprises both an afferent sensoric component and an efferent motoric component^38^. Oropharyngolarygeal sensitivity can be quantified using air puffs. This was tested using a flexible endoscope combined with a pressured air pump and was termed *fiberendoscopic evaluation of swallowing and sensitivity testing* (FEESST)^39,40^. Aviv et al. found a reduction in pharyngolaryngeal sensitivity using FEESST in older participants as well as in pathological states, such as stroke^41^. A new and similar approach has been described by Giraldo-Cadavid et al., who used a device called a *laryngeal esthesiometer* with a mechanism similar to that of FEESST^42^. It uses a combination of a camera, a high-precision airflow generator for stimulation, and a laser-based rangefinder to ensure a defined distance from the tested surface^42^. In our opinion, using a rangefinder would heighten the inter- and intrapatient comparability and is an advantage over FEESST. However, this device has not yet been tested for changes in older adults and is not commercially available. Our data complement Aviv et al.’s prior findings of a reduced oropharyngeal sensitivity in older adults using direct electrical stimulation of the faucial pillars.

A decline in taste and smell function is part of the normal aging process^43^. The loss of smell function is associated with a decrease of fibres in the olfactory bulb and a reduced olfactory epithelium surface area. An increase of receptor cell death and lower receptor cell regeneration have also been described^44–46^. An early loss of olfactory function has been found in neurodegenerative diseases such as Parkinson or Alzheimer’s diseases, which might precede clinical symptoms^45^. Gustatory dysfunction is far less common in older adults than olfactory disturbance is, and it is mostly attributed to drug use and infections (eg, candidiasis or upper respiratory tract infections^43^. Other effects on taste are attributed to lower saliva production (xerostomia) and poor dental hygiene^45^. As an age-related mechanism, altered functions of ion channels and receptors involved in taste function have been described^47^. Doty et al. and Hummel et al. reported a lower performance in smell tests among older adults compared to younger people^44,48^. Landis et al. were able to show a similar effect for taste using the Taste Strips test that was used in our experiment^13^. A trial by Stevens et al^15^ showed similar results using a different taste test^15^. As a side note, women performed better compared to men in smell and taste tests^13,44^. In our experiment, we found the same phenomenon for taste but not for smell.

The moderation analysis showed a moderating effect of the age variable on the interdependence of sensitivity and smell. Further testing revealed this effect was present solely in the older participants. We can only speculate about the mechanism of this result. As described above, the decline in sensitivity and smell are thought to share the same pathophysiology (ie, a loss of nerve fibres). A common degree of nerve fibre loss in the head and neck might explain this finding. Age-related changes in the neural vasculature have been described as explanations for peripheral nerve degeneration. However, this has not been systematically investigated for nerves in the head and neck^17^. Because the data on this matter are insufficient, our explanation remains speculative. An altered function of receptors and ion channels was described for taste, which might explain the missing moderation effect for taste.

Our study has some limitations. The results of the moderation analysis have to be interpreted with caution because only limited variation was observed in the group of younger participants. Since the Sniffin’ Sticks test and the Taste Strips test are designed as a screening test, a more thorough test might have shown a larger variance. A threshold test for taste and smell would have provided more detailed information regarding the relationship of the 3 modalities. A larger sample size could have conceivably shown different results. With a larger sample size, the effects of smoking on the different modalities might have also been detected. For the time being, we cannot explain the effect of sex on taste and smell, as other authors have done.

Currently, patients with COVID-19 infection seem to suffer from dysphagia, which leads to further deterioration or prolonged mechanical intubation^49^. Changes in oral sensitivity, taste, and smell due to COVID-related dysphagia can be further investigated using our proposed tests.

In conclusion, electrical threshold testing of the anterior faucial pillar proved to be a simple, safe, and accurate diagnostic measure in young and older patients. This diagnostic approach can be used for dysphagia-related research to quantify oral sensitivity (eg, in stroke patients).

## Methods

### Participants

The study was conducted at the university hospital in Giessen, Germany. Patients older than 59 were recruited as participants in the trauma surgery department, because the possibility of finding participants without concomitant diseases (ie, neurological diseases) was smallest in this collective. Patients with trauma to the head were not considered for the study. Healthy participants who were younger than 60 served as young controls. This group consisted of medical students and nurses or doctors from our department.

Participants were included in the study upon written informed consent. Exclusion criteria comprised past or present illnesses known to present with dysphagia (eg, Parkinson’s disease, prior stroke, chronic obstructive pulmonary disease, oropharyngeal tumours, ENT disorders, dementia), known impairment of smell and taste, known allergies to odorants or flavourings, or an implanted electronic device (ie, cardiac pacemaker). All methods were carried out in accordance with relevant guidelines and regulations for involving human participants in the study. All participants gave informed consent.

### Sensitivity threshold

**Figure 2:**
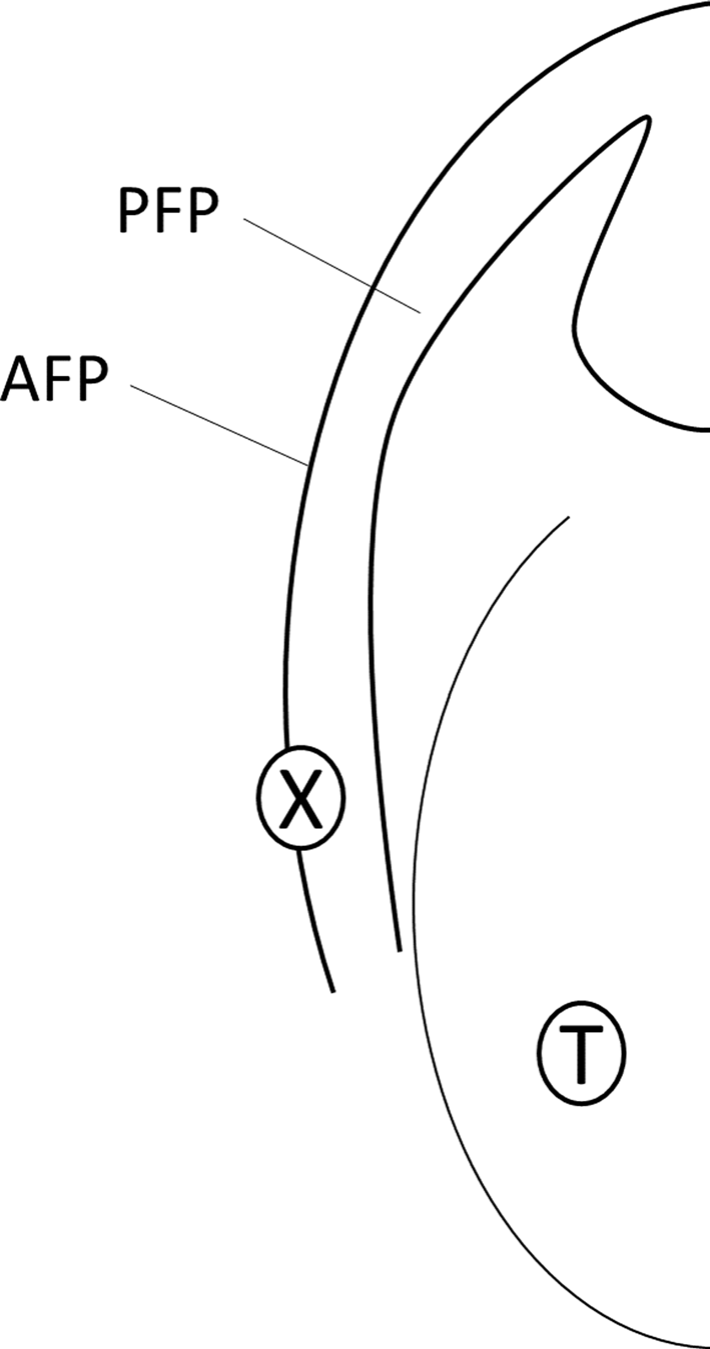
Placement of the electrode in the oral cavity. AFP: anterior faucial pillar, PFP: posterior faucial pillar, T: tongue, X: position of the anode.

A commercially available pudendal electrode was placed on the anterior faucial pillar by the examiner (having the electrode on his or her fingertip). The anode was placed with moderate pressure on the medial part of the anterior faucial pillar in the lower third just above the height of the last molar tooth. The cathode had contact with the buccal mucosa. During stimulation, the position of the fingertip was visually controlled. Figure 2 depicts the position of the anode.

The electrode was connected to a common electroneurography device. The electric stimuli were delivered with a continuous stimulation setting at a frequency of 3 Hz with a square wave duration of 200 ms. The stimulus intensity was increased in 0.2 mA steps until each participant felt the electrical sensation and the intensity in milliamps was noted. The measurement was repeated 3 times for each side in random order. For analyses, we calculated the mean values for each side as well as the mean value of both sides taken together (= total).

The stimulus intensity was not high enough to be painful or unpleasant and the stimulus was presented at the participant’s fingertips prior to oral testing. Each participant was instructed to raise his or her hand when he or she felt the stimulus.

### Taste

Taste was assessed using the commercially available Taste Strips Test (Burghart Messtechnik). A filter paper strip with a 2 cm^2^ tip was placed in the middle of each participant’s tongue. The tip was impregnated with different tastants (4 basic qualities) in 4 different concentrations. The following concentrations were used for the taste strips: sweet = 0.4, 0.2, 0.1, and 0.05 g/ml sucrose; sour = 0.3, 0.165, 0.09, and 0.05 g/ml citric acid; salty = 0.25, 0.1, 0.04, and 0.016 g/ml sodium chloride; and bitter = 0.006, 0.0024, 0.0009, and 0.0004 g/ml quinine hydrochloride. The participant had to choose the answer from the 4 different qualities (forced choice). A taste score was formed from the number of correct answers. Normgeusic values range from 9 to 16 for the sum score. Scores for the 4 different taste qualities can range from 0 to 4. Landis et al. validated the test in a large study^13^.

### Smell

Smell was tested with a commercially available screening test using 12 different felt-tip pens soaked with different odorants (Sniffin’ Sticks, Burghart Messtechnik). Each odorant was presented to the middle of a participant’s nose. The test contained aromatic and trigeminal odorants that had to be identified. For each odorant, a participant had to choose the correct answer from a list of 4 different options (forced choice). A smell score was formed from the number of correct answers. Normosmic values range from 11 to 12^48^.

### Experimental procedure

To avoid potential facilitation of the sensitivity threshold by taste or smell, the sensitivity test was performed first. We then tested the remaining modalities. The order of tests (smell first or taste first) was randomised. No side effects (eg, choking or vomiting) occurred during testing and the participants reported no gustatory perception during electrical stimulation.

### Statistical analysis

Statistical analyses were performed using SPSS version 22.0 (IBM). Variables were checked for normal distribution and outliers (defined as more than 3 SDs from the group mean). Shapiro–Wilk’s tests showed deviations from normal distribution for taste and smell scores in the young group as well as for smell score in the older group (α < 0.05). Because each group consisted of more than 15 participants and they were even in number, and because analysis of variance (ANOVA) has been shown to be relatively robust against violation of normality assumption in simulation studies^50^, we decided to proceed with the calculation of ANOVAs. We did not find outliers except for 2 young-group participants’ smell scores, which is ascribed to the small variance in smell function in this group. Because their smell scores (9) were still well within the range of possible values, we did not exclude nor transform these outliers. For the investigation of differences between groups, we calculated ANCOVAs using sensitivity threshold, smell score, and taste score (as well as sub-scores of the different taste qualities sweet, salty, sour, and bitter) as outcomes for separate analyses. Our independent variable was age, which is conceptualized as group differences. Furthermore, habitual smoking (1 = *yes*; 0 = *no*) and sex (1 = *male*; 0 = *female*) were included as covariates in the models. For the analysis of interdependence between sensitivity, taste (as well as taste quality sub-scores), and smell, we conducted multiple regression analyses (again, using group as well as habitual smoking and sex as covariates). The regression models had no autocorrelation because the value of the Durbin-Watson statistic was between 1.57 and 2.19. Concerning the influence of age on these associations, a moderation analysis was conducted using the PROCESS macro for SPSS^51^.

In 1 young person, we were unable to measure the sensitivity threshold of the left side, as well as the sensitivity threshold on the right side in 1 older adult, even when increasing the intensity to 100 mA. Their data was excluded from further analyses. No values were missing in any of the remaining variables.

### Ethics approval and consent to participate

For the data acquisition and the use of findings for scientific analyses, ethical approval was obtained from the local ethics committee (Justus-Liebig University, Protocol No. 149/16).

## Data Availability

The authors declare that the data supporting the findings of this study are available within the article.

## Abbreviation

FEESST: Fiberendoscopic evaluation of swallowing and sensitivity testing
